# Local field potentials get funny

**DOI:** 10.1113/JP272673

**Published:** 2016-07-01

**Authors:** Matthew F. Nolan

**Affiliations:** ^1^ Centre for Integrative PhysiologyUniversity of EdinburghEdinburghEH8 9XDUK

The local field potential (LFP) is widely used experimentally to index cortical activity and information processing. Classic studies using recordings of LFPs include the first demonstration of long‐term potentiation (Bliss & Lomo, 1973) and descriptions of oscillatory brain states (Buzsaki & Draguhn, 2004), while more recent applications extend as far as control of brain–machine interfaces by indirect monitoring of single neurone activity (Hall *et al*. 2014). The power of LFP recordings comes from both their high temporal and spatial resolution, and the relative ease with which they can be obtained.

Surprisingly, given their long‐standing importance, it is only relatively recently that the ionic mechanisms that determine the waveform of cortical LFPs have begun to be extensively scrutinized (Buzsaki *et al*. 2012). For a long time LFPs were often assumed to primarily reflect currents through synaptic receptors in the membrane of pyramidal cells. However, membrane currents are also carried by voltage‐gated ion channels. These active membrane conductances are responsible for generation of action potentials and for integration of synaptic responses. Recent modelling studies suggest important influences on the LFP of voltage‐gated Na^+^ and K^+^ channels that mediate action potential firing (Reimann *et al*. 2013), but the contributions of ion channels that mediate synaptic integration at membrane potentials below the action potential threshold have remained unclear.

In this issue of *The Journal of Physiology*, Ness *et al*. (2016) address the influence of subthreshold ion channel signalling on the LFP. They begin by considering the LFP generated in a detailed model of a pyramidal neurone responding to a single synaptic input. They show that when the neurone's membrane potential is −80 mV, which is likely to correspond to its resting state, the LFP response following a synaptic input to the neurone's apical dendrite depends on active conductances in the neurone's membrane. When active conductances are present, the duration of the LFP response is reduced and the amplitude of the LFP response measured near to the soma is attenuated. In contrast, for synaptic inputs to the soma, or for apical synaptic inputs arriving when the neurone's membrane potential is depolarized to −60 mV, the presence of active conductances has very little effect on the LFP response. Thus, active conductances may make important contributions to LFPs driven by dendritic synaptic inputs when neurones are in their resting state.

Which ionic current mediates these effects? Ness *et al*. demonstrate a key role for the hyperpolarization‐activated current (*I*_h_) found in the apical dendrites of pyramidal neurones. *I*_h_ is a mixed Na^+^ and K^+^ current that is activated by membrane potential hyperpolarization. In contrast most other voltage‐gated ion channels are activated by depolarization. This distinct property of *I*_h_ led to early studies referring to it as the ‘funny’ current. Because of its unusual voltage dependence, *I*_h_ opposes changes in membrane potential; when a neurone is hyperpolarized, activation of *I*_h_ causes an opposing depolarization, and vice versa when a neurone is depolarized. Because its activation kinetics are relatively slow compared to the membrane time constant of a typical neurone, *I*_h_ is most effective at opposing slow changes in membrane potential. This restorative action causes the appearance of resonance when responses to inputs with waveforms of different frequencies are considered (e.g. Nolan *et al*. 2004). Ness *et al*. demonstrate that this resonant effect, which was previously observed when recording membrane potential, is also present in LFP responses. Thus, when neurones are in their resting state and input is to their apical dendrites, *I*_h_ attenuates LFP responses with frequencies below approximately 10 Hz. Further analysis by Ness *et al*. demonstrates that this effect relies on the voltage‐dependent properties of *I*_h_ and is greatest when inputs target a single dendritic region and the channels mediating *I*_h_ are in the dendrites close to the input.

What implications do these results have for experiments measuring the LFP? When LFPs are used to assess synaptic strength, as is the case for many investigations of long‐term potentiation, the results suggest that changes in the duration of LFP responses could be attributable to active membrane conductances. For investigation of oscillatory network activity, the results of Ness *et al*. predict that when signals originating in synaptic input to apical dendrites are recorded extracellularly at sites near to the soma, their components with frequency less than approximately 10 Hz will be attenuated by *I*_h_. From this perspective, it is intriguing that genetic deletion of *HCN1*, one of the major contributors to *I*_h_ in pyramidal neurones, causes an increase in the amplitude of theta (4–9 Hz) frequency oscillations recorded from somatic regions (Nolan *et al*. 2004). This corresponds well to the predictions made by Ness *et al*. Nevertheless, these predictions rest on modelling of contributions of single neurones to the LFP. Critical issues still to be addressed include the extent to which *I*_h_, or other active subthreshold conductances, influences LFPs in network models containing many neurones, and identification of the network activity states in which these influences are most apparent.

In summary, Ness *et al*. identify a previously unappreciated role for subthreshold active conductances in shaping the local field potential. This may be particularly relevant to experimental investigation of both evoked synaptic responses and oscillatory network activity including theta and other lower frequency network oscillations.
